# Hepatic stress associated with pathologies characterized by disturbed glucose production

**DOI:** 10.15698/cst2019.03.179

**Published:** 2019-01-28

**Authors:** Monika Gjorgjieva, Gilles Mithieux, Fabienne Rajas

**Affiliations:** 1 Institut National de la Santé et de la Recherche Médicale, U1213, Lyon, F-69008, France.; 2 Université de Lyon, Lyon, F-69008 France.; 3 Université Lyon I, Villeurbanne, F-69622 France.

**Keywords:** glucose-6 phosphate, diabetes, glycogen storage disease type I, glucotoxicity, lipotoxicity, NAFLD, hepatic tumors

## Abstract

The liver is an organ with many facets, including a role in energy production and metabolic balance, detoxification and extraordinary capacity of regeneration. Hepatic glucose production plays a crucial role in the maintenance of normal glucose levels in the organism i.e. between 0.7 to 1.1 g/l. The loss of this function leads to a rare genetic metabolic disease named glycogen storage disease type I (GSDI), characterized by severe hypoglycemia during short fasts. On the contrary, type 2 diabetes is characterized by chronic hyperglycemia, partly due to an overproduction of glucose by the liver. Indeed, diabetes is characterized by increased uptake/production of glucose by hepatocytes, leading to the activation of *de novo* lipogenesis and the development of a non-alcoholic fatty liver disease. In GSDI, the accumulation of glucose-6 phosphate, which cannot be hydrolyzed into glucose, leads to an increase of glycogen stores and the development of hepatic steatosis. Thus, in these pathologies, hepatocytes are subjected to cellular stress mainly induced by glucotoxicity and lipotoxicity. In this review, we have compared hepatic cellular stress induced in type 2 diabetes and GSDI, especially oxidative stress, autophagy deregulation, and ER-stress. In addition, both GSDI and diabetic patients are prone to the development of hepatocellular adenomas (HCA) that occur on a fatty liver in the absence of cirrhosis. These HCA can further acquire malignant traits and transform into hepatocellular carcinoma. This process of tumorigenesis highlights the importance of an optimal metabolic control in both GSDI and diabetic patients in order to prevent, or at least to restrain, tumorigenic activity during disturbed glucose metabolism pathologies.

## LIVER FUNCTION IN GLUCOSE PRODUCTION AND NORMOGLYCEMIA

Maintaining normal glucose levels in the organism i.e. between 0.7 to 1.1 g/L, is a complex task, involving a multi-organ crosstalk responsible for metabolic homeostasis. This molecular machinery is crucial for the normal functioning of the body, given that glucose is considered one of the main metabolites ensuring energy production in the cells. “Energy source” has always been the main function attributed to glucose. However, glucose plays other essential roles in the cell, such as providing carbon skeletons on which all other specialized biochemical pathways ultimately depend [1]. Indeed, glucose can be a limiting factor in cell proliferation not only by its energetic role, but above all by providing carbons for nucleotide synthesis via the pentose phosphate pathway (PPP), required for DNA replication. Thus, the various roles of glucose impose a strict regulation of the concentration of this metabolite in the organism, while aberrations in the maintenance of normoglycemia can induce deleterious phenotypes and pathological states.

### Glucose production during fasting periods

During fasting periods, glucose is consumed by all organs, resulting in a decrease in glucose levels. Normoglycemia is maintained by endogenous glucose production (EGP), a process occurring in the liver, as well as in the kidneys and in the intestine [1]. EGP is activated by glucagon or counter-regulatory hormones such as epinephrine or norepinephrine. Hepatic EGP relies on two different pathways: glycogenolysis and gluconeogenesis (GNG). Renal and intestinal EGPs rely only on GNG. Short-term fasts induce the hepatic glycogenolysis pathway, entailing a degradation of glycogen into glucose-6 phosphate (G6P), which is further hydrolyzed into glucose. Once hepatic glycogen stores are depleted during prolonged fasts, hepatocytes activate GNG and thus use amino acids, lactate, pyruvate and glycerol as substrates in order to synthetize glucose *de novo*. Therefore, glycogenolysis and GNG use different substrates for glucose production, yet share the last reaction – the hydrolysis of G6P into free glucose and inorganic phosphate by glucose-6 phosphatase (G6Pase). While hepatic glucose production is a widely known process, renal and intestinal GNG has been often neglected, even though they contribute immensely to glycaemia homeostasis during long-term fasting. Indeed, during 24 h – 48 h fasting, kidney and intestine can be responsible for up to 50% and 20% of glucose production, respectively, as shown in rodents, which corroborates comparable results in humans [1–6]. In addition, intestinal GNG plays a central regulatory role in energy homeostasis in the post-absorptive state. By delivering glucose directly in the portal vein, intestinal GNG induces a gut-brain glucose signal that positively controls different metabolic functions, such as food intake and insulin sensitivity [1]. Failure to activate these physiological pathways due to metabolite imbalance or improper signalization caused by genetic, nutritional or environmental reasons can result in hypoglycemia.

### Glucose storage during postprandial/post-absorptive periods

After the ingestion of a meal, different nutrients such as sugars, lipids and proteins are digested and absorbed. The absorption of glucose raises the circulating concentration of this metabolite in the blood stream. While glucose is essential for the normal functioning of the organism, excessive amounts can induce glucotoxicity [7, 8]. In order to prevent glucotoxicity and to form glucose stores needed during fasting, glucose is captured by the liver and the peripheral tissues and stored under the form of glycogen and lipids. This process is orchestrated by insulin, which is known to activate glycogen synthesis, lipogenesis, as well as protein synthesis. Failure to restore normoglycemia after meals entails hyperglycemia and diabetes.

**Figure 1:**
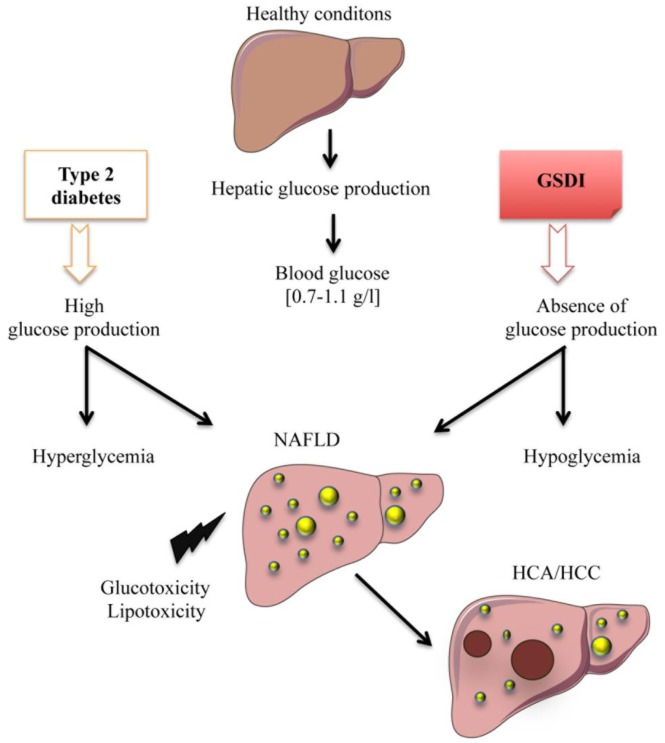
FIGURE 1: Deregulation of endogenous glucose production leads to hepatic complications. In normal physiological conditions, the liver maintains glucose homeostasis in the blood by releasing free glucose during fasts and by up-taking and storing excessive glucose during post-prandial periods. In type 2 diabetes, hepatic production and uptake of glucose is deregulated, resulting in hyperglycemia. In Glycogen Storage Disease type I (GSDI), glucose production in completely abolished, resulting in hypoglycemia. Strikingly, both of these pathologies are characterized by non-alcoholic fatty liver disease (NAFLD) development that can lead to hepatocellular carcinoma in some cases.

In this review, we will focus on the cellular stress induced in the liver under two different pathophysiological states linked to deregulation in EGP, inducing either chronic hyperglycemia (Type 2 diabetes) or hypoglycemia (Glycogen Storage Disease type I – GSDI). In both cases, this deregulation leads to the development of fatty liver disease and, in some patients, it can lead to hepatic tumor development (**Figure 1**).

## DEREGULATION IN ENDOGENOUS GLUCOSE PRODUCTION AND FATTY LIVER

**Figure 2:**
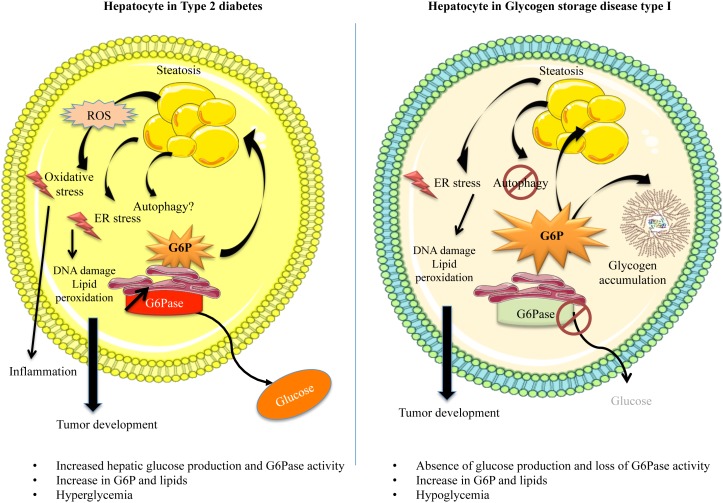
FIGURE 2: Dysfunction of hepatocyte metabolism in type 2 diabetes and GSDI leads to cell stress. Type 2 diabetes is associated with hyperglycemia partially due to an overproduction of glucose by the liver, since G6Pase activity is increased, whereas GSDI is associated with hypoglycemia due to the absence of G6Pase activity. However, these two diseases share similar hepatic metabolism leading to the development of fatty liver. In type 2 diabetes, hyperglycemia leads to an increase of the metabolic flux downstream of G6P, whereas in GSDI, the absence of G6Pase activity is responsible for G6P accumulation. In both diseases, this results in an activation of *de novo* lipogenesis. In addition, GSDI is characterized by glycogen accumulation. These metabolic perturbations are responsible for cell stress such as ER stress. Even though mitochondrial dysfunctions were shown in both diseases, increased ROS production and oxidative stress has only been observed in diabetes, but not in GSDI. In GSDI, autophagy is also clearly decreased, but this is still controversial in diabetes. In both diabetes and GSDI, cell stress could cause DNA and protein damages, lipid peroxidation and finally, the development of hepatic tumors.

Type 2 diabetes is the most common condition characterized by chronic hyperglycemia. With the expansion of the western civilization lifestyle, the incidence of this disease, along with obesity, has dramatically risen worldwide [9]. Type 2 diabetes is characterized by insulin resistance of the tissues capable of capturing glucose and/or the lack of insulin production due to β–cell function decline [10]. As mentioned, chronically elevated glucose levels in the bloodstream can induce glucotoxicity. Most studies in diabetes-associated glucotoxicity address this phenomenon in the β–cells, as a negative retroactive system amplifying insulin secretion dysfunction. However, in the liver, dissociating the exact mechanisms behind cell stress induced by glucose toxicity or by lipid toxicity, such as in diabetic and/or obese patients is very difficult. Glucose and lipid metabolisms are tightly linked, due to the interchange of common metabolites. Thus, diabetes, characterized by glucose metabolism dysfunction, is linked to liver disease, more precisely to non-alcoholic fatty liver disease (NAFLD) [11, 12]. Indeed, up to 70% of diabetic patients may present NAFLD [11, 12]. Even if glucose uptake is impaired in obese/diabetic mice [13], elevated levels of blood glucose induce an increased metabolic flux downstream of G6P and a subsequent activation of *de novo* lipogenesis (**Figure 2**) [14]. Insulin and glucose can both induce hepatic lipogenesis. Interestingly, insulin-mediated lipogenesis is activated via sterol regulatory element–binding protein-1c (SREBP-1c), while glucose-mediated lipogenesis is activated via carbohydrate response element binding protein (ChREBP) [15, 16]. More precisely, glucose metabolites such as G6P or xylulose 5-phosphate were suggested to directly activate ChREBP [15]. SREBP-1c and ChREBP are key transcription factors in lipogenesis [17]. Paradoxically, even in insulin-resistant states, this hormone still manages to activate hepatic lipogenesis *via* SREBP-1c. *De novo* lipogenesis is not the only process contributing to fatty liver. Indeed, increase in hepatic lipid storage also results from diet, elevated non-esterified fatty acids (NEFA) due to a decreased inhibition of adipose tissue lipolysis, reduced hepatic lipid oxidation, as well as reduced lipid export in the form of very low density lipoprotein (VLDL) [18]. Finally, hepatic lipids tend to further accentuate insulin resistance by interfering with insulin signaling, thus enclosing a vicious cycle.

While hyperglycemia is widely spread and classified as an epidemic, chronic hypoglycemia (plasma glucose level lower than 0.55 g/L) is not a very common condition, although acute hypoglycemic episodes are often experienced in diabetic patients undergoing unstable therapy or receiving excessive amounts of insulin. Interestingly, patients with GSDI, a rare genetic disease (1/100,000 live births) suffer from chronic hypoglycemia during short fasting periods [19]. Indeed, as opposed to type 2 diabetes, this pathology is characterized by an absence of EGP due to a deficiency in G6Pase (**Figure 1** and **2**) [19–21]. This leads to the accumulation of G6P in hepatocytes. As in diabetic patients (see above), increased G6P activates glycogen synthesis and *de novo* lipogenesis, leading to hepatomegaly and severe steatosis induced by strong glycogen and lipid accumulation, respectively (**Figure 1** and **2**), associated with hypercholesterolemia and hypertriglyceridemia [22, 23]. In GSDI human livers, *de novo* lipogenesis and cholesterol synthesis were found to be increased 40-fold and 7-fold, respectively [24]. Furthermore, conversion of VLDL into intermediate density lipoproteins is delayed. Lipid vesicles are present in abundance in GSDI livers, mainly in the periportal zone, which corresponds to the location of the highest expression of G6Pase in the liver [25]. In addition, high G6P levels induce glycolysis and PPP, leading to lactic acidosis and hyperuricemia, respectively [26–28].

Before developing the molecular events in regards to cell stress in diabetic and GSDI hepatocytes, it is noteworthy that both of these pathologies are characterized by an increase of the metabolic flux downstream of G6P [29]. This elevation leads to a deep metabolic reprogramming, which induces cell stress in the liver of both diabetic and GSDI patients [26].

## OXIDATIVE STRESS IN THE CONTEXT OF HYPERGLYCEMIA AND HEPATIC STEATOSIS

The glucotoxic and lipotoxic effects in the liver of diabetic subjects have many facets. Oxidative stress is one of the mechanisms behind this toxicity in the liver [30–33], but it presents as a multi-organ pathology that can also be responsible for β–cell loss-of-function, vascular complications and strokes, neuropathy, retinopathy and nephropathy in diabetes [34].

The mechanisms of G6P-derived cell stress include increased polyol pathway flux, increased intracellular formation of advanced glycation end-products (AGEs), activation of protein kinase C (PKC), increased hexosamine pathway or overproduction of superoxides by the mitochondrial electron transport chain [35–38]. The polyol pathway leads to the conversion of glucose to sorbitol and further to fructose, thus reducing the availability of cofactors such as NADPH needed for the glutathione peroxidase–glutathione reductase system, rendering the cell more vulnerable to reactive oxygen species (ROS), and therefore reinforcing oxidative stress [39, 40]. Furthermore, during chronic hyperglycemia, glucose can be autooxidized and form covalent adducts with the plasma proteins through a non-enzymatic process known as glycation, leading to the formation of AGEs [41–43]. Glycation of proteins interferes with their normal functions by disrupting molecular conformation, altering enzymatic activity and interfering with receptors' function. AGEs interact with receptors for AGEs (RAGE) to alter intracellular signaling, gene expression, release of pro-inflammatory molecules and free radicals. Finally, the increase in the hexosamine pathway flux is a complex process involving the usage of glucose to produce glucosamine-6-phosphate and subsequently UDP-N-acetylglucosamine (UDP-GlcNAc), resulting in pro-fibrotic signalization, characterized by the induction of transforming growth factor β1 (TGF-β1) and plasminogen activator inhibitor-1 (PAI1) expression [44, 45].

Oxidative stress is due to an increased amount of ROS in the cell that can result from decreased antioxidant activity and/or increased ROS production. ROS notably include superoxide anion (O^-^_2_), hydrogen peroxide (H_2_O_2_) and hydroxyl radical (HO^-^). ROS production is mainly attributed to the mitochondrial complexes in the electron transport chain, responsible for the oxidative phosphorylation, as well as NADPH oxidases, xanthine oxidase, nitric oxide synthase. The production of these highly unstable molecules is a physiological process that is tightly regulated by antioxidant activity, in order to prevent the negative cellular outcome of ROS. However, in pathological conditions, antioxidant activity can be exceeded or insufficient to restore the physiological concentration of ROS and thus lead to oxidative stress. Indeed, during cell stress and/or mitochondrial insult, mitochondrial complex I and III mainly contribute to oxidative stress by producing O^-^_2_ [46]. In addition, membrane-bound NADPH oxidases can be activated by inflammatory signals and can also lead to the production of O^-^_2._ Diabetic animals present decreased hepatic activity of antioxidant enzymes such as catalase and superoxide dismutase 1, leading to increased ROS and hydroperoxides [47, 48]. The increase in ROS levels can damage lipids, proteins, DNA, RNA and can affect the functioning of various organelles in the cell, such as the mitochondria or the endoplasmic reticulum (ER), thus leading to a pathological state in the hepatocytes [32, 48]. For example, lipid peroxidation is a process where ROS can interact with the lipid's electrons and thus alter its structure and characteristics, leading to important disturbances in the bi-layer lipid membranes of the cell, which can have strong effects on cell homeostasis and signalization, as well as survival. Taking into consideration that type 2 diabetes is associated with hepatic lipid accumulation, oxidative stress represents a major issue in liver function in these patients. Furthermore, ROS can easily target the reduction state of sulphurcontaining amino acids, cysteine (Cys) and methionine (Met) with great impact on the protein structure and/or enzymatic activity. Hydroxylation and carboxylation of proteins mediated by ROS can also occur during oxidative stress. Moreover, the quantification of the latter is often used to assess the extent of oxidative damage in the cells [49]. Protein damage and aggregation can easily impact the functioning of the ER and thus induce ER stress. In regards to DNA, ROS can induce structural modifications of the bases, inter- and intra-strand crosslinks, induce strand breaks and promote DNA-protein crosslinks [50]. Indeed, oxidative stress-induced DNA damage has been documented in diabetes [51]. Last, ROS overproduction leads to depletion in adenosine triphosphate (ATP) and nicotinamide dinucleotide (NAD), which directly affects energy homeostasis in the cell [52].

### ROS and lipids

While the lipid storage capacity of adipose tissues is much higher than other tissues, the liver is able to store large amount of lipids in form of triglyceride (TG), diacylglycerol and cholesterol esters [53]. Hepatic lipids accumulate in cytoplasmic lipid droplets and form highly dynamic organelles also containing proteins, such as perilipin [54, 55]. Many studies suggest that TG, even in high amounts in the liver, when stored in lipid vesicles, are relatively inert, non-reactive and thus rather inoffensive. While the storage of NEFA in form of TG represents a protective mechanism [56], their excessive accumulation and lipid droplet enlargement may lead to cell damage [57, 58]. Indeed, NEFA spillover or the inability to store further NEFA in TG due to lack of capacity to further enlarge lipid droplets and the accumulation of specific lipotoxic compounds could lead to increased oxidative stress. Thus, lipids like ceramides, diacylglycerol and phosphatidic acid were shown to contribute strongly to insulin resistance and therefore, to amplify the diabetic phenotype [59, 60]. Furthermore, free cholesterol and fatty acids are attracting more and more attention as the designated cell injury drivers in hepatic lipid-related pathologies, due to their important reactivity with other components of the cell. First, increased presence of fatty acids in the liver can induce the production of free radicals. Indeed, the oxidation of fatty acids in the mitochondria, peroxisomes and microsomes, mediated in part by cytochrome P450 (CYP2E1), CYP4A10, and CYP4A14, results in an increase in ROS [16, 61]. Conversely, ROS are responsible for increased lipid peroxidation. Trans-4-hydroxy-2-nonenal (HNE) and malondialdehyde (MDA) are some of the most studied lipid peroxidation products that can be highly toxic in the cells. These molecules can interact with DNA and form etheno-DNA adducts, leading to carcinogenesis [62]. Moreover, aberrant lipid metabolism in diabetic/obese patients can lead to an induction of ER stress [63]. Indeed, hepatic lipid metabolism deregulation was shown to alter the composition of the phospholipids within the ER and thus perturb protein synthesis [64]. Moreover, protein aggregation in lipotoxic conditions can also induce this process. While ER stress can be induced by lipid misbalance, this process has an important pivotal role on lipid metabolism as a whole. For example, forced expression of BiP, a key negative regulator of the ER stress response, was shown to protect against hepatic steatosis by inhibiting SREBP1c mediated lipogenesis [65]. Indeed, all of the components of the ER stress response, which includes the IRE1a/XBP1 axis, the PERK/ATF4 axis and ATF6 have all been shown to promote lipogenesis when activated, mainly by promoting SREBP1c expression, but also directly increasing the expression of lipogenic genes such as FAS, ACC and SCD1 [65]. As expected, attenuating ER stress in obese rodents decreases steatosis and improves insulin sensitivity [66].

### Oxidative stress and inflammation

Oxidative stress results in an increase in apoptosis of hepatocytes and a subsequent release of inflammatory cytokines, attracting infiltration of the liver by inflammation-mediated leukocytes [67]. Pro-inflammatory mediators involved in hyperglycemic liver damage include interleukins such as IL-1 and IL-6, nuclear factor (NF-kB), mitogen-activated protein *kinase* (MAPK), TGF-β1, poly ADP-ribose polymerase (PARP) and tumor necrosis factor-α (TNFα) [31, 68–70]. Indeed, diabetic rat models confirmed that in the liver, the induction of TNFα results in increased levels of NF-kB and JNK signaling, characterized by further induction of inducible nitric oxide synthase (iNOS) and consequent increase in nitric oxide production, as well as increased apoptosis rates [71]. Specific inhibition of TNFα resulted in a decrease in the before-mentioned pathways, as well as decreased apoptosis [71]. Treatment with antioxidants such as Tempol also prevented lipid peroxidation and apoptosis induced by TNFα and iNOS and the subsequent oxidative stress [72]. On the other hand, increased circulating levels of Il-6 were found to be responsible for the activation of another inflammatory pathway (STAT3-dependent) in the liver [73, 74]. While Il-6 is a mitogen required for efficient regeneration of the liver, chronic Il-6 activation could have a strong impact on the hepatocytes and can be involved in the development of a pathological state. Strikingly, other studies have shown that Il-6 can possibly have an anti-inflammatory role in diabetes, confirming that the molecular signalization in diabetes is quite complex [75].

The production of these inflammatory factors is due to various cell types infiltrating the liver, such as neutrophils, macrophages and T-lymphocytes, depending on the stage and condition of the hepatic pathology [76]. Furthermore, resident cells in the liver, such as Kupffer cells (macrophages) and even hepatocytes can also play a major role in inflammation mediation. Interestingly, inflammation seems to play a key role in promoting the progression of NAFLD to steatohepatitis and further to cirrhosis and cancer. For example, significant infiltration of T cells is detected in patients with NAFLD and correlates with the disease severity, suggesting that T cells promote the progression of NAFLD [77]. In addition, it was shown that metabolic changes linked to NAFLD promote a selective inhibition of CD4^+^ T lymphocytes infiltration, while CD8^+^ lymphocytes were unaffected, leading to an acceleration of hepatic carcinogenesis [78]. Thus, the activation of inflammatory cells in the liver leads to hepatocyte injury and liver fibrosis by producing ROS and inflammatory mediators (as described above). However, inflammation can also have beneficial effects by stimulating removal of dead cells and liver regeneration. Thus, inflammatory cells and mediators in the liver could have multifaceted functions in the liver by promoting pathogenesis progression of NAFLD or protecting hepatocytes against apoptosis, as described by Gao and Tsukamoto (2016) [76]. Finally, inflammation can also have a direct role on insulin sensitivity. Indeed, ROS can inhibit insulin signaling by inducing Insulin Receptor Substrate (IRS) degradation in peripheral tissues, thus leading to insulin desensitization [79]. Similarly, TNFα was also shown to induce insulin resistance.

## HEPATIC OXIDATIVE STRESS AND INFLAMMATORY STATUS IN GSDI

While deciphering the clinical aspects of GSDI is a highly important task, this pathology has much to offer to basic research as well. Indeed, GSDI is characterized by chronic hypoglycemia in the absence of treatment, yet in the hepatocytes these patients present extreme metabolic features comparable to those observed in diabetes. Paradoxically, GSDI patients present increased risk of insulin resistance even though they suffer from hypoglycemia, when they are not under optimal nutritional care [80]. In order to study GSDI, several mouse models have been developed, including total deletion and liver-specific deletion of the gene encoding the catalytic subunit of the G6Pase (*G6pc*) [81, 82]. Total deletion mouse models present severe hypoglycemia especially in the absence of oral or injected glucose, leading to premature death in these mice after weaning. However, liver-specific *G6pc*-deficient (L.G6pc-/-) mice are viable, rendering this model particularly well suited for long-term studies. Indeed, L.G6pc-/-mice can produce glucose from their kidneys and intestine during fasting [83]. Both of these models develop the hepatic pathology observed in GSDI patients, including hepatomegaly and hepatic steatosis, associated with hypercholesterolemia and hypertriglyceridemia [81, 82].

### Mitochondrial dysfunction and ROS

Mouse models have generated interesting data in regards to cell stress associated with GSDI. As previously mentioned, G6Pase deficiency leads to hepatocyte metabolism characterized by the activation of glycolysis, *de novo* lipogenesis, PPP and glycogen synthesis [22, 23]. Interestingly, hepatic mitochondrial dysfunction was reported, along with a striking decrease in basal respiration, ATP turnover, maximal respiration, and spare mitochondrial capacity [84]. The structure of mitochondria was abnormal and the mitochondrial content was also decreased, probably due to decreased biogenesis. Another study confirmed this result in L.G6pc-/-mice, showing that lipid-mediated Sirtuin1 (SirT1) down-regulation entails a decrease in peroxisome proliferator-activated receptor-γ coactivator 1α (PGC-1α), and thus alters mitochondrial integrity, biogenesis, and function in GSDI hepatocytes [85]. The mitochondrial apoptosis pathway is also activated [84]. Indeed, an increase in cytochrome c release, as well as activation of caspases 9 and 3 were reported in *G6pc*-knock down cells. Finally, mitochondrial dysfunction was linked to insulin resistance [80]. Despite this pathological mitochondrial phenotype, ROS levels were not increased in the cells, leaving room to speculate that oxidative stress might not be present in the case of GSDI. Furthermore, increased circulating levels of antioxidants reported in GSDI patients could contribute to the protection against oxidative stress [86]. Elevated circulating antioxidants could also protect GSDI patients against atherosclerosis, despite hyperlipidemia [87, 88]. It is note-worthy that hyperuricemia, albeit a pathological state, can also provide antioxidant defense, since plasma uric acid is also a potent low-molecular-weight antioxidant. However, within the cell, uric acid can have pro-oxidative roles as well, by forming radicals with other oxidants, rendering the effect of this metabolite in GSDI complex [89].

Since in GSDI the capacity of G6P storage under the form of glycogen is chronically exceeded, G6P activates *de novo* lipogenesis and leads to hepatic steatosis. Hepatic steatosis in GSDI is also enhanced by a decrease in lipid β–oxidation. This catabolic pathway was shown to be down-regulated in the liver of L.G6pc-/-mice [90], with a concomitant down-regulation of the main activator of β–oxidation, PPARα. It has been suggested that the production of malonyl CoA by acetyl CoA carboxylase (ACC) during lipogenesis could further contribute to the decrease in β–oxidation in GSDI livers. Interestingly, a reactivation of PPARα in the liver of L.G6pc-/-mice via its agonist, fenofibrate, resulted in a normalization of the hepatic TG content and thus a complete disappearance of hepatic steatosis in these mice [91]. Strikingly, fenofibrate treatment resulted in a normalization of the glycogen content in L.G6pc-/-mice as well. Finally, a decreased activity of AMP-activated protein kinase (AMPK) in GSDI hepatocytes might also contribute to impaired fatty acid oxidation and increased fatty acid and cholesterol synthesis [92]. AMPK regulates these processes by decreasing malonyl CoA production via ACC inhibition and via the control of SREBP1 and ChREBP activities. A decrease in β–oxidation could thus contribute to the absence of increased ROS in GSDI livers.

### Autophagy

Interestingly, altered lipid metabolism affects autophagy in GSDI hepatocytes (**Figure 2**). Indeed, lipid accumulation due to increased ChREBP and decreased PPARα results in a decreased SIRT1/FOXO signaling and thus in the absence of autophagy activation [93]. Since SIRT1 is down-regulated during lipogenesis, it entails a vicious cycle between lipid accumulation and autophagy in GSDI. Indeed, SIRT1 is blocked due to lipid synthesis, which subsequently blocks autophagy and leads to further lipid accumulation. Besides lipids, other metabolites, proteins and even dysfunctional organelles remain non-recycled and lead to cell stress or even contribute to malignancy. In accordance, the reactivation of autophagy pathway in GSDI resulted in an increase in lipid degradation, associated with an improved hepatic histology [92]. It is noteworthy that in L.G6pc-/-mice autophagy was found activated in the hepatic tumors, compared to the surrounding non-tumoral tissue [27]. As observed in many cancer types, this activation of autophagy in GSDI tumors could facilitate their progression by providing malignant cells with substrates for rapid proliferation, as well as a protective role against cell necrosis and inflammation [94].

Autophagy is a process that is considered as regulated by mTOR, AMPK and SIRT1 [95]. In the case of GSDI, autophagy was shown to be independent from mTOR signaling, since mTOR inhibition using Temsirolimus did not lead to autophagy activation in L.G6pc-/-mice [27]. As mentioned earlier, AMPK is strongly down-regulated in GSDI livers due to their energetic state, leaving only SIRT1 as a master regulator.

As observed in GSDI, NAFLD and diabetes can also be characterized by a decrease in autophagy. However, contradictory results showing ER stress-mediated induction of autophagy in obesity, rather than a decrease, have been reported as well. This highlighted that the levels of insulin resistance, steatosis and the overall state of the hepatocytes have a role to play in the outcome of this process [96]. Interestingly, one of the metabolites by which autophagy decrease in obesity can be mediated is nitric oxide. Indeed, obesity promotes S-nitrosylation of lysosomal proteins in the liver, thereby impairing lysosomal enzyme activities, and further facilitating hepatic steatosis and insulin resistance [97]. The canonical pathways regulating autophagy are involved in autophagy repression as well. Indeed, over-nutrition provides increased availability of amino acids and glucose in obesity, which can constitutively activate mTOR and inhibit AMPK, resulting in autophagy repression [98]. Lipids, as in GSDI, can also contribute to autophagy inhibition. However, as lipids constitute a great family of molecules with different attributes, their differential effects on autophagy can vary greatly. For example, oleic acid was shown to induce autophagy, whereas palmitic acid suppresses this process [99]. Thus while in GSDI autophagy was proven to be systematically repressed, this is not always true in diabetes and obesity, probably due to the variability in the etiology, the staging of the pathology and the variable environment in diabetic and/or obese patients.

### Inflammatory status in GSDI livers

Despite the important levels of accumulated glycogen and lipids in the liver, as well as the important metabolic imbalance, GSDI patients present low-grade hepatic inflammation [19, 100]. However, a significant elevation of serum Il-8 levels was reported in patients bearing tumors, positively correlating with neutrophilia and hepatic neutrophil infiltration [100]. Hepatic transaminase (AST/ALT) levels are also usually normal, especially in patients with optimal metabolic control and patients not bearing hepatic tumors [100].

The absence of oxidative stress and inflammatory responses in the case of GSDI might be the reason as to why hepatic fibrosis is not associated with GSDI [19]. Indeed, GSDI patients and related animal models do not present fibrosis in the liver, contrarily to other types of glycogen storage diseases. Consequently, these patients do not develop cirrhosis. However, they present a highly elevated risk of hepatic malignancy, characterized by a specific tumorigenic process described below.

## HEPATIC CARCIOGENESIS IN DIABETES AND GSDI

Nowadays it is becoming more and more evident that diabetes is associated with chronic liver disease [101], leading to an important risk of hepatocellular carcinoma (HCC) development [78–80]. Interestingly, it was suggested that similar pathways are activated in both diabetes and hepatocellular cancer [102, 105]. For example, the Insulin/Insulin Growth factor 1 (IGF1) signalization pathway and the subsequent activation of mTOR are increased in both cases. In hyperinsulinemic conditions, insulin exerts a mitogenic role, rather than a metabolic role, which is highly beneficial for HCC progression [106]. Furthermore, the aforementioned inflammatory mediators such as TNFα and Il-6 can also contribute to hepatic cancer development. Last, taking into account that cancer cells are often highly dependent on free glucose fueling the Warburg effect, chronic hyperglycemia is ideal for their rapid progression. In addition, hyperglycemia was shown to induce nuclear β–catenin accumulation in cancer cells, which could be yet another trigger of tumorigenesis in diabetes [107].

**Figure 3:**
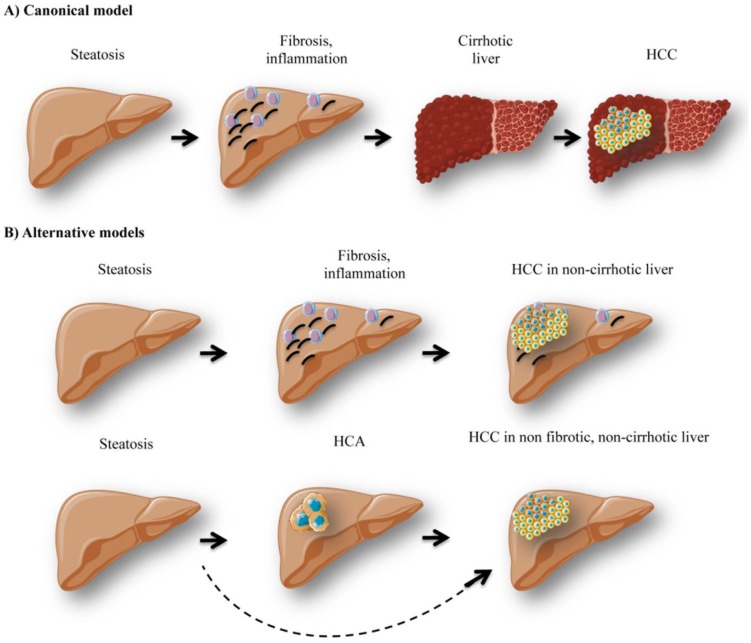
FIGURE 3: Different models of hepatocellular carcinoma development. The canonical model of hepatocellular carcinoma (HCC) development (A) stipulates that patients with hepatic steatosis further develop inflammation / immune cell infiltration and fibrosis. Later on, excessive fibrosis and inflammation can lead to cirrhosis development and HCC. However, alternative models of hepatocarcinogenesis (B) have been observed in obese / GSDI patients. Indeed, steatotic patients, who do not present cirrhosis, can also develop HCC *de novo*, since fatty livers are favorable for carcinogenesis. Moreover, in GSDI patients, HCC can develop in non-fibrotic, non-cirrhotic liver. These tumors arise from the transformation of hepatocellular adenoma (HCA) to HCC.

Interestingly, lipid-mediated expression of TNFα and oxidative stress are responsible not only for cell injury, inflammation, necrosis, but also for activation of stellate cells inducing fibrosis [108, 109]. Indeed, in the livers exposed to chronic injury, stellate cells promote the development of fibrosis through excessive extracellular matrix (ECM) production and reduced ECM degradation [110]. Reduced adiponectin levels can also potentiate the fibrogenic process [111]. Fibrosis is a common end point to chronic inflammation in insulin-resistant livers and it can be further stimulated by Kupffer cells, the resident hepatic macrophages. The formation of Mallory-Denk bodies, composed of misfolded intermediate filaments, ubiquitin, heat shock proteins, and p62, can be observed during fibrogenesis [112]. Hepatic fibrosis can further evolve to cirrhosis and HCC development (**Figure 3**). Linking cirrhosis and diabetes is very complex since cirrhosis itself is linked to insulin resistance [113]. Indeed, around 30% of patients with hepatic cirrhosis present diabetes, while cirrhosis is not necessarily induced by obesity / diabetes [114].

It is noteworthy that an important fraction of obese patients develop HCC in the absence of liver cirrhosis as well [115, 116]. Indeed, around 54% of NAFLD patients diagnosed with HCC were not classified as cirrhotic, as opposed to only 22% of Hepatitis C virus (HCV) patients [117]. Thus hyperglycemic/hyperlipidemic conditions in obesity could favor hepatic hyperplasia development that can acquire malignant traits in the absence of cirrhosis and transform into HCC (**Figure 3B**). HCC can also arise *de novo* due to extensive DNA damage and mutations occurring as a result of chronic oxidative stress. Therefore, clinical surveillance of the liver in obese patients is recommended even in the absence of fibrosis/cirrhosis, in order to successfully prevent hepatic malignancy.

GSDI patients also present an increased risk of hepatic tumor development. Indeed, around 50% of young adult patients present at least one hepatocellular adenoma (HCA) [118]. There is a high risk (about 10%) of transformation of HCA into HCC, and this rate is significantly higher in GSDI patients compared to non-GSDI patients. As opposed to HCC patients in the general population, hepatic fibrosis/cirrhosis is absent in GSDI, and therefore *de novo* formation of HCC has never been reported. Indeed, all HCC probably arise from a malignant transformation of HCA into HCC (**Figure 3B**). Thus, malignancy development in GSDI is a very particular linear process, characterized by hepatic steatosis installation, followed by HCA formation, which can later transform into HCC. The exact mechanisms behind this elevated tumor incidence in GSDI are not fully understood. However, the metabolic context in the liver of GSDI patients and animal models could provide a favorable environment for tumorigenesis [26]. As mentioned before, increased glycolysis and subsequent lactate production, elevated lipogenesis and PPP are just some of the metabolic alterations observed in GSDI livers. These metabolic traits are associated to the Warburg effect, a metabolic process infamously affiliated with cancer. Thus in GSDI, the liver itself is characterized by a cancer-like metabolism, potentially facilitating tumor formation and progression.

## FINAL REMARKS

### Metabolism and regeneration

As discussed above, the effects of glycemic imbalance are mostly studied in the central nervous system, the pancreas, in retinopathies and nephropathies, yet, hepatic damage assessment is often overlooked. This is in part due to the exceptional plasticity of the liver and the extraordinary detoxification and regeneration mechanisms that it possesses. Indeed, this organ is capable of efficient regeneration after resection. This process has fascinated mankind since the beginning of medical research, and it has been extensively studied. Some of the facets of liver regeneration remain unknown; however, it has been shown that metabolic aspects are highly important.

Indeed, liver regeneration after partial resection depicts perfectly the plasticity of the metabolism of the liver and how metabolic switches can be crucial in pathophysiology. For example, transient hepatic steatosis appearing right after resection has been reported and described as indispensable for proper regeneration and proliferation of the liver, in order for the hepatocytes to repopulate the liver [119]. In contrast, under these conditions glucose homeostasis is understandably disturbed, since the regenerating liver cannot completely assure the role of the main glucose-producing organ. This depends on the extent to which the organ is resected, the physiopathological condition of the remaining liver, as well as the overall health state of the patient. Thus, regeneration of the liver can lead to hypoglycemia, as often confirmed in many rodent models and patients with partial hepatectomy [120]. Interestingly, supplementing the liver with glucose during this phase can have a negative impact on regeneration [120, 121]. Given that hypoglycemia is thought to induce lipolysis in peripheral organs, facilitating the induction of transient hepatic steatosis needed for regeneration, this outcome is expected, yet it renders post-operative patient care complex. Moreover, hepatic ischemic episodes have been shown to alter glucose metabolism in the liver by switching from oxidative phosphorylation to a more proliferativecompatible metabolism, characterized by activation of glycolysis (the Warburg effect).

### Antioxidants against hyperglycemic damages and hepatic tumors

Since hyperglycemia is strongly associated with oxidative stress, the use of antioxidants in diabetes or in prevention or curative strategies for HCC constituted tempting approaches of treatment. Interestingly, various antioxidant agents such as metformin, Nfr2 agonists, Vitamin C and E, resveratrol, as well as different plant extracts have been used in HCC patients and patients at risk of HCC. The outcomes in these strategies varied greatly among the studies and were described as both pro- and anti-oncogenic [122, 123]. While increased ROS in the cell can be responsible for serious alterations of the DNA and other cell components, it is noteworthy that these molecules are important signaling agents, physiologically needed for the activation of certain defenses in the cell under pre-pathological conditions. Thus it seems important to emphasize that preventing this signalization with antioxidants could be harmful, rather than beneficial. As antioxidants are widely popular in the general population and not only in scientific circles, several misconceptions have been previously highlighted [124]. Indeed, the quantity, the type of antioxidants and the duration of the treatment may have an enormous impact on the outcome for the patient.

While many studies depict the effects of antioxidants in diabetic/obese patients, assessment of the effects of these drugs in GSDI patients is nearly impossible to perform, firstly because of the small number of patient cohorts, but also due to the various treatments that these patients receive in parallel, such as hypolipidemic and hypouricemic agents. However, a study in L.G6pc-/-mice using the anti-oxidant Tempol showed that while this treatment managed to increase the hepatic expression of Catalase and GPx1, it did not have an impact on carcinogenesis [27].

Last, studies have shown that under some circumstances cancer cells can also be more sensitive to oxidative stress than the surrounding healthy cells [125]. Therefore, inducing oxidative stress in tumor cells is also an attractive strategy to combat tumor progression [126]. To conclude, the redox levels in cancer and the surrounding healthy tissue can vary greatly and one unique approach is not applicable in all patients.

## Abbreviations:

AGE: advanced glycation end-product,
ChREBP: carbohydrate response element binding protein,
ECM: extracellular matrix,
EGP: endogenous glucose production,
G6P: glucose-6-phosphate,
G6Pase: glucose-6-phosphatase,
GNG: gluconeogenesis,
GSDI: glycogen storage disease type I,
HCA: hepatocellular adenoma,
HCC: hepatocellular carcinoma,
iNOS: inducible nitric oxide synthase,
NAFLD: non-alcoholic fatty liver disease,
NEFA: non-esterified fatty acid,
PPP: pentose phosphate pathway,
ROS: reactive oxygen species,
SREBP-1c: sterole regulatory element binding protein-1c,
TG: triglyceride,
TNFα: tumor necrosis factor alpha,
VLDL: very low density lipoprotein.
